# Mitochondria-Targeted Antioxidants and Skeletal Muscle Function

**DOI:** 10.3390/antiox7080107

**Published:** 2018-08-08

**Authors:** Sophie C. Broome, Jonathan S. T. Woodhead, Troy L. Merry

**Affiliations:** 1 Discipline of Nutrition, Faculty of Medical and Health Sciences, The University of Auckland, Auckland 1023, New Zealand; s.broome@auckland.ac.nz (S.C.B.); j.woodhead@auckland.ac.nz (J.S.T.W.); 2 Maurice Wilkins Centre for Molecular Biodiscovery, The University of Auckland, Auckland 1023, New Zealand

**Keywords:** mitochondria, reactive oxygen species, oxidative stress, skeletal muscle, antioxidant

## Abstract

One of the main sources of reactive oxygen species (ROS) in skeletal muscle is the mitochondria. Prolonged or very high ROS exposure causes oxidative damage, which can be deleterious to muscle function, and as such, there is growing interest in targeting antioxidants to the mitochondria in an effort to prevent or treat muscle dysfunction and damage associated with disease and injury. Paradoxically, however, ROS also act as important signalling molecules in controlling cellular homeostasis, and therefore caution must be taken when supplementing with antioxidants. It is possible that mitochondria-targeted antioxidants may limit oxidative stress without suppressing ROS from non-mitochondrial sources that might be important for cell signalling. Therefore, in this review, we summarise literature relating to the effect of mitochondria-targeted antioxidants on skeletal muscle function. Overall, mitochondria-targeted antioxidants appear to exert beneficial effects on mitochondrial capacity and function, insulin sensitivity and age-related declines in muscle function. However, it seems that this is dependent on the type of mitochondrial-trageted antioxidant employed, and its specific mechanism of action, rather than simply targeting to the mitochondria.

## 1. Introduction

Reactive oxygen species (ROS) are continuously produced by skeletal muscle from a number of mitochondrial and non-mitochondrial sources, with production being increased during contractile activity [1]. Skeletal muscle fibers contain a well-developed endogenous antioxidant defence network consisting of the primary antioxidant enzymes superoxide dismutase (SOD), glutathione peroxidase (GPX) and catalase, in addition to other antioxidant enzymes such as thioredoxins, glutaredoxins and peroxiredoxins, and nonenzymatic antioxidants, such as glutathione [1]. Under most conditions, these cellular antioxidants work as a complex unit to regulate ROS, maintain oxidative balance and protect cells against oxidative damage. However, prolonged exposure to high levels of ROS can overwhelm the antioxidant defense system leading to oxidative damage of proteins, nucleic acids and lipids, resulting in cellular dysfunction, and has been associated with the pathogenesis of muscle related diseases such as type 2 diabetes, cachexia, and several dystrophies [2,3,4,5], as well as impaired exercise performance and recovery [1,6,7]. For this reason, the use of oral antioxidant supplements to support the endogenous antioxidant defence system has received much attention as a potential strategy to limit oxidative stress and promote muscle health and performance [1,7,8]. Somewhat paradoxically, ROS are increasingly being recognised as important signalling molecules that regulate skeletal muscle function and adaptation, and are required for optimal cell functioning [9,10,11,12]. As such, it is probably not surprising that supplementation with non-targeted antioxidants have had little impact on disease development and progression [9,13,14,15] and exercise performance [16,17] and in some instances have been reported to be deleterious [18,19,20,21]. This has driven the development of inhibitors [4,11,22,23] and cellular organelle-targeted antioxidants that are aimed at decreasing ROS levels from specific production sites in the cell. 

It is clear that skeletal muscle produces ROS from numerous subcellular sites in a controlled and regulated manner in response to physiological stimuli, and may become unregulated under pathophysiological stimuli. The mitochondria have been cited as the major site of superoxide production in skeletal muscle, with approximately 0.15% of the oxygen consumed by the mitochondria undergoing one electron reduction to generate superoxide [24]. A number of potential alternative sites of ROS production have been identified including nicotinamide adenine dinucleotide phosphate (NADPH) oxidases, xanthine oxidases, and phospholipase A2 dependent processes. It is beyond the scope of this review to discuss the production of ROS from these non-mitochondrial sources, and readers are directed to the following comprehensive reviews [1,25]. The production of ROS from these non-mitochondrial sources appears to be linked to the signalling processes that modulate skeletal muscle adaptation, whereas excess mitochondrial ROS are more often associated with oxidative damage and disease states [26,27,28]. There has been extensive research focusing on transgenic mice with targeted overexpression of the human catalase gene to mitochondria, which has highlighted the role of mitochondrial ROS production in numerous disease states [29,30,31]. As a result, mitochondria-targeted antioxidants are increasingly being developed as a potential strategy to limit mitochondrial ROS production and oxidative damage, potentially without suppressing ROS that are important for signalling, and are becoming more widely available as an over the counter supplement. Compared with general antioxidants, mitochondria-targeted antioxidants are chemically modified to enable their transport across biological membranes where they accumulate several-hundred fold within the mitochondria and decrease ROS [32]. The purpose of this review is to discuss the literature that relates to exogenous mitochondria-targeted products that primarily act as antioxidants, specifically, MitoQ (10-(4,5-dimethoxy-2-methyl-3,6-dioxo-1,4-cyclohexadien-1-yl)decyl) triphenylphosphonium mesylate), and SkQ1 (10-(6′-plastoquinonyl) decyltriphenylphosphonium), which are antioxidants that are targeted to the mitochondria, as well as the mitochondria locating peptides SS-31 (d-Arg-2′, 6′-dimethyltyrosine-Lys-Phe-NH_2_) and XJB-5-131, and their effects on muscle function.

## 2. Targeting Antioxidants to the Mitochondria

### 2.1. Conjugation of Antioxidants to Lipophilic Cations

Antioxidants such as ubiquinone and plastoquinone have been targeted to the mitochondria through conjugation to a lipophilic cation, such as the triphenylphosphonium (TPP^+^) cation, which can pass easily through phospholipid bilayers. The ability of these positively charged cations to cross the plasma membrane allows them to accumulate substantially within the mitochondria owing to the large membrane potential [33,34]. As the plasma membrane potential is approximately 30–60 mV (negative inside), lipophilic cations accumulate 5–10 fold within the cytoplasm. The mitochondrial membrane potential is typically 140–180 mV, therefore the cations further accumulate several hundredfold within the mitochondria. It is important to consider that accumulation of the lipophilic cation within the mitochondria can result in the dissipation of the mitochondrial membrane potential when targeting antioxidants to the mitochondria using lipophilic cations, as this may have implications for their transport into the mitochondria and, thus, impact on their therapeutic capacity. 

**Figure 1:**
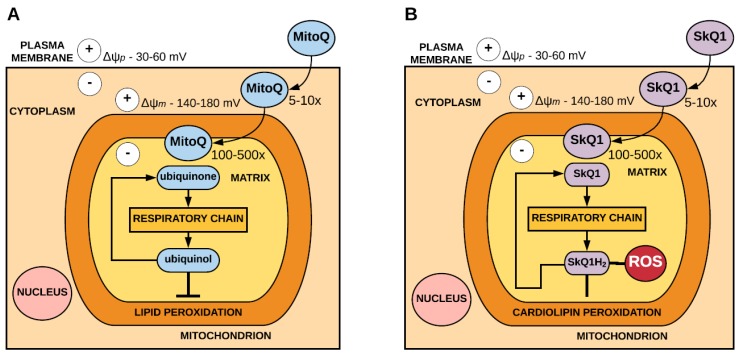
Accumulation and antioxidant mechanism of 10-(4,5-dimethoxy-2-methyl-3,6-dioxo-1,4-cyclohexadien-1-yl)decyl) triphenylphosphonium mesylate (MitoQ) (**A**) and 10-(6′-plastoquinonyl) decyltriphenylphosphonium (SkQ1) (**B**). Driven by the plasma membrane potential (Δψ*_p_*), MitoQ and SkQ1 pass through the plasma membrane and accumulate 5-10 fold within the cytosol. MitoQ and SkQ1 then accumulate several-hundredfold within the mitochondria driven by the mitochondrial membrane potential (Δψ*_m_*).Within the mitochondria, MitoQ is reduced to ubiquinol and SkQ1 is reduced to SkQH_2_ by the respiratory chain. In acting as an antioxidant, ubiquinol is oxidised to ubiquinone and SkQH_2_ is oxidised to SkQ1, both of which are rereduced by the respiratory chain. MitoQ is effective at preventing lipid peroxidation. SkQ1 is effective at preventing peroxidation of cardiolipin and inhibits the production of superoxide. (adapted from Murphy and Smith, 2007 [39]).

MitoQ, which consists of a ubiquinone moiety, is targeted to the mitochondria by conjugation to the TPP^+^ cation. Within the mitochondria, MitoQ localises to the inner mitochondrial membrane and is reduced to the active antioxidant ubiquinol by complex II of the respiratory chain. In acting as an antioxidant, the ubiquinol form of MitoQ is oxidised to form ubiquinone, which is then reduced by complex II to ubiquinol. MitoQ is an effective antioxidant against lipid peroxidation and has also been shown to detoxify peroxynitrite [35] (Figure 1). 

Similarly, SkQ1, which comprises a plastoquinone moiety, is also targeted to the mitochondria via the conjugation of the TPP^+^ cation [36]. Within the mitochondria, SkQ1 is reduced to SkQ1H_2_ by the respiratory chain. SkQ1 acts as an antioxidant by preventing peroxidation of the mitochondrial phospholipid cardiolipin [37] and inhibiting the formation of superoxide [38]. In acting as an antioxidant SkQH_2_ is oxidised to SkQ1, which is reduced by the respiratory chain to SkQH_2_ (Figure 1).

### 2.2. Mitochondria-Targeted Peptides that Incorporate Antioxidants

**Figure 2:**
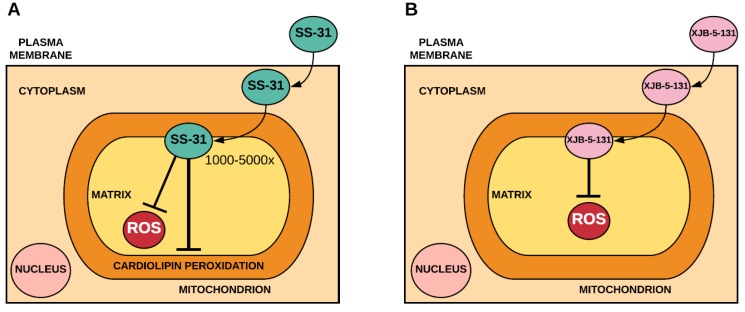
Accumulation and antioxidant mechanism of Szeto-Schiller-31 (SS-31) (**A**) and XJB-5-131 (**B**). SS-31 accumulates several-thousand fold within the mitochondria where it binds to cardiolipin. In acting as an antioxidant, it is likely that SS-31 prevents peroxidation of cardiolipin and decreases mitochondrial ROS production. XJB-5-131 accumulates within the mitochondria independent of the membrane potential, where it acts as an antioxidant by scavenging mitochondrial ROS.

Szeto-Schiller (SS) peptides are a series of antioxidant peptides that contain an amino acid sequence, which allows them to pass through plasma membranes independent of the membrane potential and localise to the inner mitochondrial membrane [40]. Data suggests that SS-31 targets cardiolipin [41] and that its antioxidant mechanism likely involves decreased mitochondrial ROS production rather than direct savaging of ROS [42] (Figure 2). In addition to SS peptides, several peptides that consist of an electron and ROS scavenger (4-NH_2_-TEMPO) conjugated to fragments of the gramicidin S cyclopeptide antibiotic, known as XJB peptides, have been used to limit oxidative stress [43,44] (Figure 2).

## 3. Mitochondrial Biogenesis and Function

General antioxidants have been shown to attenuate mitochondrial biogenesis, particularly in response to exercise [19,20,21]. However, the ROS that signal the initiation of mitochondrial biogenesis during exercise may not be coming from the mitochondria. Indeed, exercise has been shown to increase skeletal muscle mitochondrial capacity during concurrent MitoQ supplementation [45], which supports previous studies that suggest that non-mitochondrial ROS production plays a central role in mediating exercise training-induced adaptations [1,24,46,47]. Similarly to exercise, high-fat diets have been reported to initiate mitochondrial biogenesis via a ROS dependent activation of CaMKII [48]. During high-fat feeding, mitochondrial biogenesis may promote a higher reliance on fatty acids as a fuel source and may actually decrease mitochondrial ROS production by dissipating proton-motive force over a greater mitochondrial volume. This potentially compensatory effect can be reversed by SkQ1 administration [48], which suggests that mitochondria-targeted antioxidants may not be beneficial under all conditions of metabolic stress. This is supported by the finding that high fat diet-induced mitochondrial dysfunction was not improved following SkQ1 administration [49]. In contrast, however, MitoQ has been shown to restore mitochondrial function during high-fat feeding [50], which suggests that the effect of mitochondrial-targeted antioxidants on mitochondrial function during high-fat feeding may be dependent on a number of factors including its mechanism of action, dose, and the duration and type of metabolic stress.

## 4. Insulin Sensitivity

Transient increases in ROS, such as those that occur during exercise, have been implicated in the promotion of insulin sensitivity, and general antioxidants have been shown to attenuate the improvements in insulin signalling and sensitivity that are associated with acute and chronic (training) exercise [20,51]. On the contrary, high levels of mitochondrial ROS production and oxidative stress have been implicated in the development of insulin resistance [26,52]. As such, the effect of mitochondria-targeted antioxidants on insulin sensitivity during times of increased oxidative stress, such as during high-fat feeding, is being increasingly investigated. SS-31 preserved insulin sensitivity in mice fed a high-fat diet [26], and MitoQ has been shown to partially reverse high-fat diet-induced glucose intolerance [50,53]. This suggests that clearance of mitochondrial ROS through the use of mitochondria-targeted antioxidants is associated with improved glucose homeostasis under conditions of metabolic stress. However, whilst SkQ1 treatment attenuated high fat diet-induced oxidative stress, there was no effect on glucose tolerance or insulin signalling [49]. This again suggests that the effect of the mitochondria-targeted antioxidant employed on insulin sensitivity during high-fat feeding is dependent on the specific mechanism of action rather than simply mitochondrial targeting, the dose used or possibly the duration of the high-fat diet, and likely highlights the multifaceted nature of mechanisms underpinning insulin resistance. 

## 5. Skeletal Muscle Contractile Function

Physiological levels of ROS that are present under basal conditions are essential for normal force production. However, changes in the redox state of skeletal muscle can have a significant effect on force production, fatigue development and recovery [1,6,7]. Several authors have reported that acute antioxidant supplementation delays muscle fatigue during highly fatiguing exercise [54,55,56,57]. However, ROS that are responsible for the decrease in skeletal muscle contractile function that occurs during fatiguing stimulation potentially come from non-mitochondrial sources. Indeed, SS-31 had no effect on force production during fatiguing stimulation in isolated muscle fibres [58], nor did it affect fatigue-induced decreases in contractile force [59]. During recovery, SS-31 restored the fatigue-induced decrease in sarcoplasmic Ca^2+^ release but did not improve force recovery in isolated muscle fibres [59]. The specific action of the mitochondria-targeted antioxidant employed appears to be an important factor in a number of other parameters of muscle function. Therefore, more studies implementing other mitochondria-targeted antioxidants are needed to further substantiate these findings.

## 6. Ageing, Sarcopenia and Disuse Muscle Atrophy

Ageing is associated with a progressive decline in muscular function and the development of diseases such as sarcopenia (muscle loss) and mitochondrial dysfunction [60]. The free-radical theory of ageing states that these changes may be driven by an increased production of ROS [61,62]. Levels of mitochondrial ROS have been shown to increase with age and have been suggested to damage mitochondria resulting in lower adenosine triphosphate (ATP) production and mitochondrial respiration capacity [30]. These shifts are hypothesised to contribute to the metabolically dysfunctional phenotype (muscular atrophy and decreased cellular respiration) seen with ageing [30,63]. Therefore, targeting mitochondrial ROS production with mitochondria-targeted antioxidants may be an effective strategy to prevent muscular dysfunction associated with ageing. 

Mitochondria-targeted antioxidants have been shown to exert largely beneficial effects on measures of muscular function with age. SS-31, XJB-5-131, and SkQ1 have shown promise in protecting/restoring muscle from the ageing phenotype [64,65,66]. However, disappointingly MitoQ has failed to attenuate age-related decline in muscle function of mice, but this may relate to no observable effects on muscle oxidative balance at the doses given [67]. Whereas, SS-31 and XJB-5-131 supplementation have been reported to increase respiratory complex activity (CI, III, and IV) and muscle fiber contractile properties (indicating maintained protein quality) in aged rodents in comparison to age-matched controls [65,66], however, whether this translated to improvements in vivo health and life span remains unclear. Additionally, SS-31 supplementation restores time to muscular fatigue, ATP production capacity, oxidative phosphorylation (phosphate/oxygen ratio), and energy state (PCr/ATP ratio) to levels comparable to that of younger mice, 1 hour following a single dose. Extended treatment with SS-31 further showed significantly lowered mitochondrial H_2_O_2_ emissions, indicating enhanced redox status, which is thought to be contributing to the restoration of mitochondrial function. Therefore, early research suggests great promise for these mitochondria-targeted antioxidants as potential supplements to attenuate aged related functional decline. 

Oxidative damage is also associated with muscular atrophy in ageing, and interestingly, SkQ1 supplementation has been shown to decrease pathological changes in mitochondrial structure including myofibril structural retardation and autophagosome accumulation in 24-month old rats. Supplemented rats suffered less degradation of mitochondrial reticulum and cristae structures, which are involved in maintaining electron transport chain activity and ATP synthesis capacity [68]. Similar findings are seen in muscular atrophy models utilising limb immobilisation, where increased mitochondrial oxidative damage associates with muscle atrophy [64,69,70]. Mice with immobilised hind-limbs and supplemented with SS-31 had reduced H_2_O_2_ production which was comparable to age-matched ambulatory controls. This effect also translated to a retention of muscle to body-weight ratio, muscle fiber cross-sectional area and mitochondrial energetic state activity similar to that of ambulatory controls. 

Taken together, targeted inhibition of mitochondrial sources of ROS by SS-31, SkQ1, and XJB-5-131 have shown promising restorative and protective effects that could be implemented in ageing and immobilization-induced muscular atrophy/dysfunction therapies. However, these findings have largely been demonstrated in rodent models and, therefore, there is a clear need for human clinical trials investigating the effects of these antioxidants within an ageing and muscle wasting context.

## 7. Mitochondria-Targeted Antioxidants in the Clinic

In general, mitochondria-targeted antioxidants have been shown to exert beneficial effects on muscle function through the improvement or attenuation of declines in mitochondrial capacity and function, insulin sensitivity and age or immobilisation induced-atrophy (Table 1). These findings suggest that mitochondria-targeted antioxidants may be a useful therapy for skeletal muscle related diseases that involve mitochondrial ROS production and oxidative damage. However, to date, studies that have investigated the effects of mitochondria-targeted antioxidants on skeletal muscle function have mostly employed rodent models, and there are very few studies that have translated these findings to a human population or clinical setting. Given that ROS are beginning to be implicated in the pathogenesis of several muscular dystrophies [5], investigating the utility of mitochondria-targeted antioxidants in the treatment of muscle diseases in a clinical setting requires further investigation. Antipodean Pharmaceuticals Inc. developed MitoQ as a pharmaceutical and now MitoQ is available as an over the counter supplement, and has been shown to be well tolerated and safe in doses up to 80 mg twice daily [71]. Originally MitoQ was investigated for its ability to act as a disease-modifying agent in newly diagnosed patients with Parkinson’s disease (PD) [71] but appeared to be ineffective in altering disease progression over 12 months. It seems likely that by the time PD is clinically evident, the fate of the remaining dopaminergic neurons is already determined and neuroprotection cannot prevent their death. In contrast, MitoQ has been shown to be effective in reducing serum alanine transaminase in patients with Hepatitis C virus (HCV) infection [72], indicating that it may be effective in reducing liver damage in HCV infection. Furthermore, MitoQ has also been shown to improve endothelial function and aortic stiffness in individuals with elevated baseline levels [73], suggesting that MitoQ and other therapeutic strategies that target mitochondrial ROS hold promise for treating age-related vascular dysfunction. To date, the results from human studies indicate that MitoQ can be safely administered to patients for up to a year [71] and that these doses are effective in decreasing liver damage and improving endothelial function. 

SS-31 has been in clinical development with Stealth BioTherapeutics Inc. since 2010 using the acetate salt form (MTP-131), and has been shown to be safe and well tolerated [74,75,76,77]. SS-31 (also referred to as elamipretide or Bendavia) was originally investigated for its ability to prevent ischemia-reperfusion injury in patients with acute ST-segment elevation myocardial infarction undergoing percutaneous coronary intervention, however, disappointingly SS-31 failed to decrease myocardial infarct size [75]. Conversely, SS-31 has been shown to attenuate the development of transient hypoxia after renal stenting in patients with atherosclerotic renal artery stenosis [78], suggesting that targeted mitochondrial protection may minimise the ischemic injury associated with such procedures. Other researchers investigating the effectiveness of SS-31 in the treatment of acute heart failure have shown that a single infusion can induce favourable changes in cardiac structure and function [76]. Furthermore, 5 days of SS-31 treatment has been shown to increase exercise performance in patients with primary mitochondrial myopathy [77]. The results from this study suggest that SS-31 improves ATP synthesis regardless of the underlying genetic defect impairing mitochondrial respiration [77]. The results from human studies involving SS-31 have been overwhelmingly positive, indicating that it may be a promising treatment in a wide range of human diseases and disorders that involve mitochondrial oxidative damage.

These results clearly highlight a role for mitochondria-targeted antioxidants in a clinical setting for the treatment of diseases involving mitochondrial oxidative damage. Future studies are needed to translate the results of animal studies indicating that mitochondria-targeted antioxidants may be an effective treatment for skeletal muscle-related diseases, such as type 2 diabetes, and age-related declines in muscle function to humans in a clinical setting.

## 8. Conclusions

**Figure 3:**
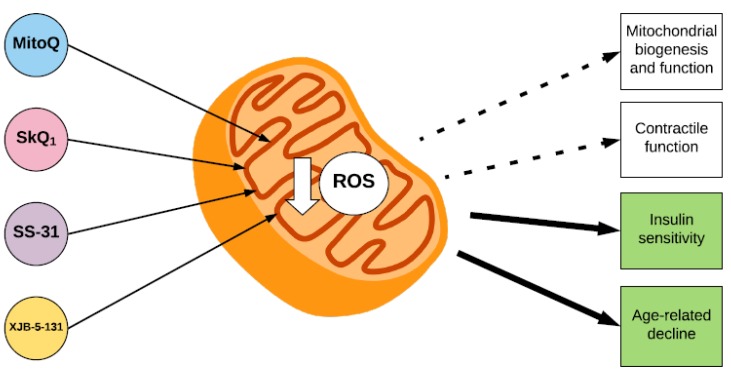
The effect of mitochondria-targeted antioxidants on parameters of skeletal muscle function. The mitochondria-targeted antioxidants MitoQ and SkQ1, and the mitochondria-locating peptides SS-31 and XJB-5-131 accumulate within the inner membrane of the mitochondrion, where they decrease ROS. Mitochondria-targeted antioxidants have a beneficial effect on insulin sensitivity and age-related declines in muscle function (indicated by green squares). However, their effects on mitochondrial biogenesis and function and skeletal muscle contractile function are still unclear (indicated by white squares).

To date, the results from human clinical trials suggest that mitochondria-targeted antioxidants, specifically MitoQ and SS-31, may be effective treatments in the pathology of a variety of human diseases that involve mitochondrial oxidative damage. In general, studies that have investigated the effect of mitochondria-targeted antioxidants on different parameters of muscle function indicate that they exert a beneficial effect on muscle function by improving or attenuating declines in mitochondrial capacity and function, atrophy, and insulin sensitivity (Figure 3). However, their effects appear to be dependent on the specific mechanism through which they limit mitochondrial ROS, the doses used, and possibly the context in which ROS production is increased. Future studies should focus on translating the findings from animal studies, which indicate that mitochondria-targeted antioxidants may be a beneficial strategy for the treatment of skeletal muscle related diseases involving mitochondrial ROS production and oxidative damage, to a human population in a clinical setting.

## Figures and Tables

**Table 1:** The effect of mitochondria-targeted antioxidants on different parameters of muscle function.

Measure Parameter	Antioxidant	Model	Supplementation Protocol	Result
Mitochondrial biogenesis and function	MitoQ	Exercise training, humans	10 mg/day	No effect [45].
		High fat diet, rats	375 µmol/kg for 8 weeks	↑ Mitochondrial function [50].
	SkQ1	High fat diet, mice	250 nmol/kg for 16 weeks	↓ Mitochondrial biogenesis [51].
		High-fat diet, rats	250 nmol/kg for 16 weeks	No effect [48].
Insulin sensitivity	MitoQ	High-fat diet, rats	375 µmol /kg for 8 weeks	↑ Glucose tolerance [50,53]
	SkQ1	High-fat diet, rats	250 nmol/kg for 16 weeks	No effect [49].
	SS-31	High-fat diet, rats	1.5 mg/kg for 6 weeks	↑ Insulin sensitivity [26].
Contractile function	SS-31	Fatiguing stimulation of isolated skeletal muscle fibers	3 µmol	No effect [58].
			200 nM	No effect [59]
				No effect [59].
Ageing, sarcopenia and disuse muscle atrophy	MitoQ	Ageing, mice	100 µmol for 15 weeks	No effect [67].
	SkQ	Ageing, rats	250 nmol/kg	↓ Age-associated pathological changes in mitochondrial structure [68].
	SS-31	Ageing, mice	3mg/kg	↑ Time to muscular fatigue, ATP production capacity, oxidative phosphorylation, and energy state [65].
		Hind limb immobilisation, rats [64] and mice [70]	3 mg/kg for 7 days [64], 1.5 mg/kg for 14 days [70]	↓ Oxidative damage, mitochondrial dysfunction and atrophy [64,70].
	XJB-5-131	Aged rats	3 mg/kg for 4 weeks	↑ Respiratory complex activity and muscle fiber contractile properties [66].
